# Supplementary material for: Optimizing cancer immunotherapy response prediction by tumor aneuploidy score and fraction of copy number alterations
Source: NPJ Precis Oncol. 2023 Jun 3;7:54. doi: 10.1038/s41698-023-00408-6 (PMC10239491; doi:10.1038/s41698-023-00408-6)
Supplement: Supplementary file 1 — Supplementary information [file 41698_2023_408_MOESM1_ESM.pdf]

**Cohort**	**Cancer type**	**HR**	**Current p value**	**Current sample size**	**Estimated sample size for p < 0.05**
**Samstein et al.**	Cancer of unknown primary	1.68	0.13	88	288
**Samstein et al.**	Colorectal cancer	1.46	0.21	110	558
**Samstein et al.**	Non-small cell lung cancer	1.19	0.19	350	1602
**Samstein et al.**	Esophagogastric cancer	1.17	0.55	126	2816
**Samstein et al.**	Head and neck cancer	1.16	0.53	139	2782
**Chowell et al.**	Hepatobiliary cancer	1.61	0.18	52	232
**Chowell et al.**	Ovarian cancer	1.36	0.48	31	502
**Chowell et al.**	Colorectal cancer	1.32	0.48	46	676
**Chowell et al.**	Mesothelioma	1.17	0.69	34	1696
**Chowell et al.**	Sarcoma	1.07	0.83	67	11040
**Chowell et al.**	Head and neck cancer	1.07	0.82	69	9366
**Chowell et al.**	Esophagogastric cancer	1.05	0.83	108	19156

Supplementary Table 1. Sample size estimates for achieving statistically significant survival difference (Kaplan-Meier HR > 1, $p < 0.05$) in individual cancer types.
The "estimated sample size for $p < 0.05$" is calculated with power analysis (see Methods).

Metric	Cancer type	Gene	Metric-high (%)	Metric-low (%)	p value	adj. p value
$AS_{0.1}$	Non-small cell lung cancer	*TP53*	71.8	51.5	0.005	1
$AS_{0.1}$	Non-small cell lung cancer	*PTPRT*	14.9	4.7	0.029	1
$AS_{0.1}$	Non-small cell lung cancer	*RB1*	11.6	2.4	0.022	1
$AS_{0.1}$	Non-small cell lung cancer	*KMT2D*	12.2	3.0	0.028	1
$AS_{0.1}$	Non-small cell lung cancer	*FAT1*	13.8	4.7	0.049	1
$AS_{0.1}$	Melanoma	*TP53*	28.1	15.6	0.049	1
$AS_{0.2}$	Non-small cell lung cancer	*TP53*	74.1	53.6	0.004	1
$AS_{0.2}$	Non-small cell lung cancer	*KEAP1*	12.6	25.1	0.037	1
$AS_{0.2}$	Non-small cell lung cancer	*RB1*	13.3	2.9	0.015	1
$AS_{0.2}$	Melanoma	*BAP1*	1.5	8.7	0.045	1
$FGA_{0.2}$	Non-small cell lung cancer	*TP53*	71.4	52.6	0.009	1
$FGA_{0.2}$	Non-small cell lung cancer	*RB1*	12.0	2.3	0.017	1
$FGA_{0.2}$	Melanoma	-	-	-	-	-

Supplementary Table 2. Differential gene mutation frequency (%) in AS (or FGA) high versus low group in melanoma and non-small cell lung cancer.
Data are from the Samstein et al. cohort.

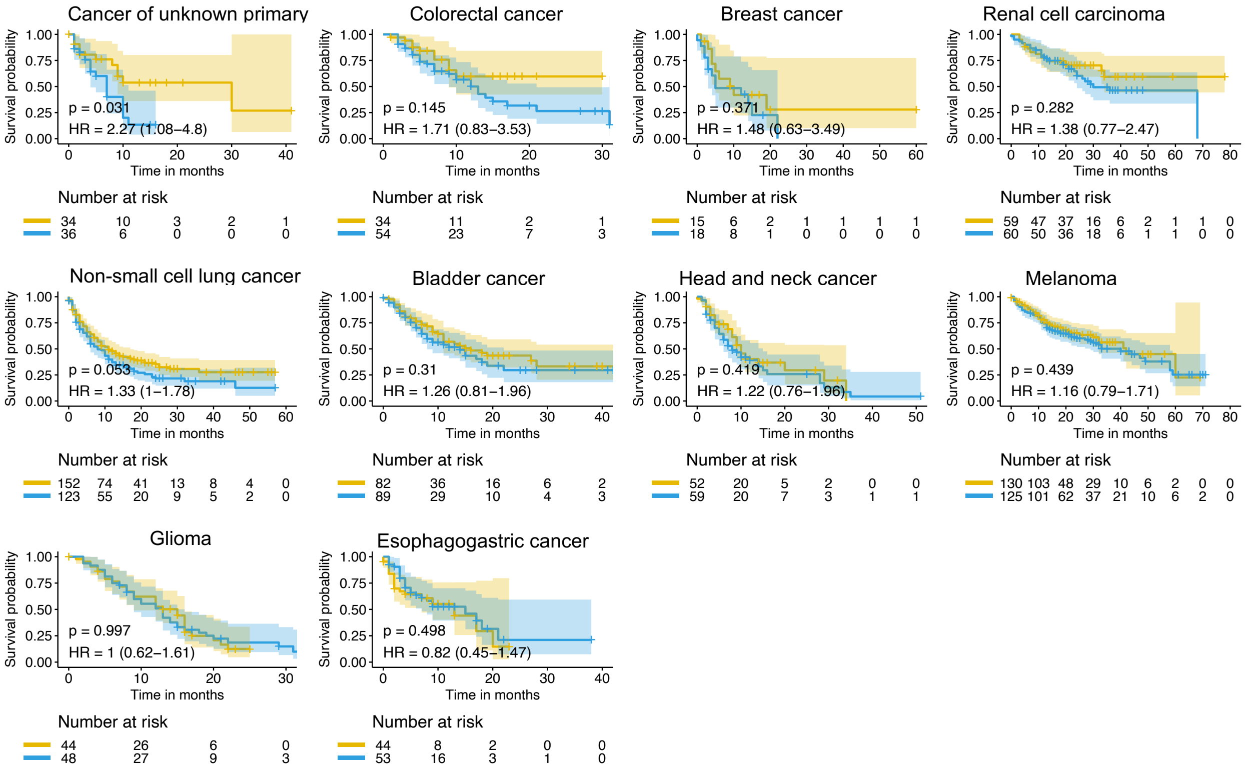

Supplementary Figure 1. Tumor aneuploidy score (AS0.1) is not significantly predictive of survival following immunotherapy in *individual* cancer types.

Kaplan-Meier survival curves following immunotherapy are compared for low-TMB patients (<80th percentile) with high versus low $AS_{0.1}$ (binned into AS-low and AS-high at the 50th percentile) across 10 individual cancer types. Univariate Cox regression HRs with 95% confidence intervals and p values are displayed.

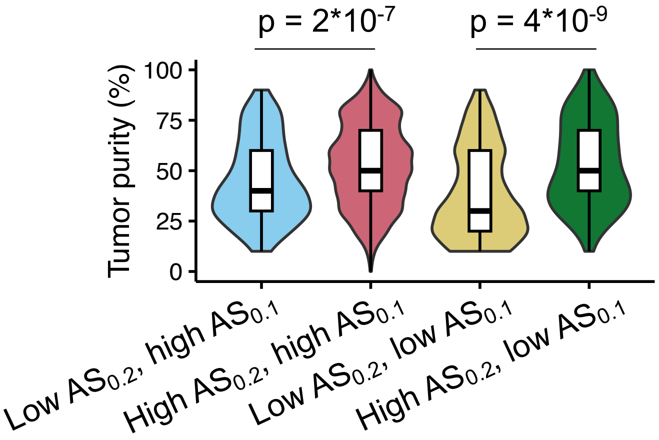

Supplementary Figure 2. Comparison of the tumor purity between different groups of pan-cancer patients based on binarized $AS_{0.1}$ and $AS_{0.2}$ scores.

Violin plots are used to depict the distributions of tumor purity in different groups. The upper and lower boundaries of the boxes represent the first and third quartiles, respectively, while the central line denotes the median. Whiskers extend to the most distant data points that are not considered outliers (within 1.5 times the interquartile range).

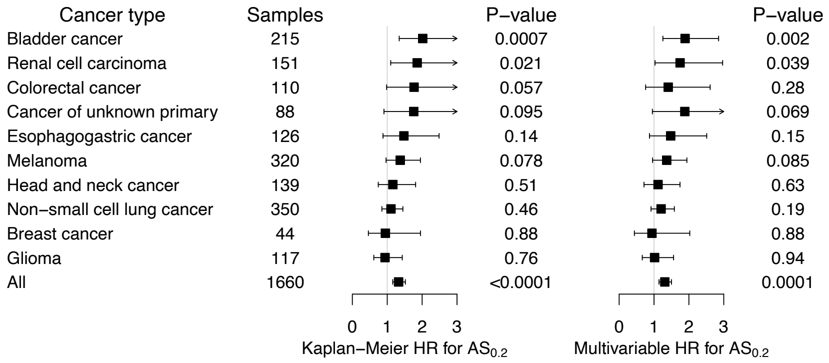

Supplementary Figure 3. Prediction of survival following immunotherapy using $AS_{0.2}$. Univariable Kaplan-Meier survival analysis and multivariable survival analysis using Cox proportional hazards regression of overall survival with $AS_{0.2}$ (binned at the 60th percentile), TMB (binned at the 80th percentile), and ICB drug class. Wald p values are displayed. Squares positioned at midpoints symbolize point estimates of HRs, and the accompanying bars indicate 95% confidence intervals. The data are from the Samstein et al. cohort [1].

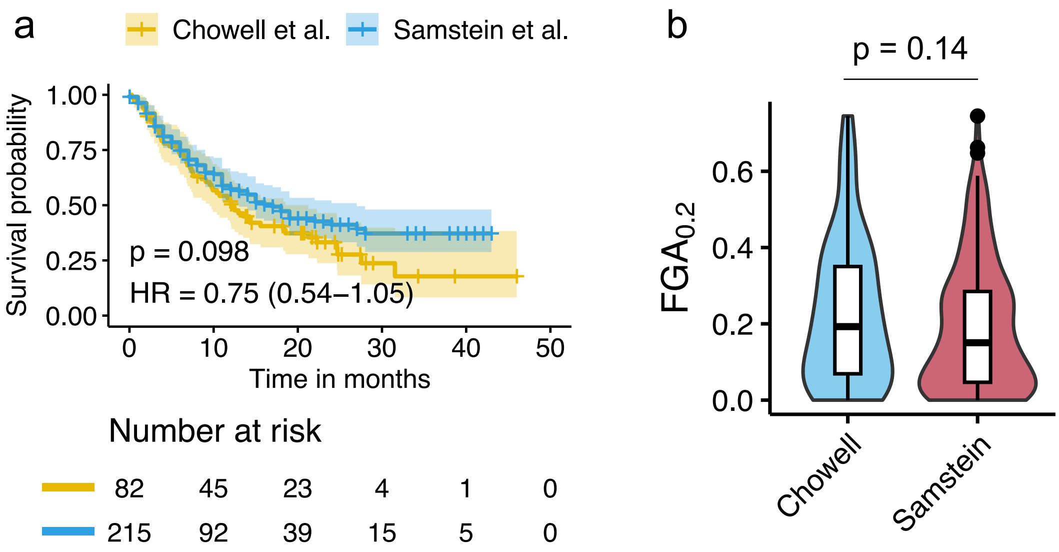

Supplementary Figure 4. Heterogeneity of bladder cancer in two cohorts.
(a) Kaplan-Meier survival curves among bladder cancer patients in the Samstein et al. cohort versus the Chowell et al. cohort. **(b)** Distribution of $FGA_{0.2}$ values of patients in the two cohorts. In panel b, violin plots are used to depict the distributions of $FGA_{0.2}$ in different cohorts. The upper and lower boundaries of the boxes represent the first and third quartiles, respectively, while the central line denotes the median. Whiskers extend to the most distant data points that are not considered outliers (within 1.5 times the interquartile range), and outliers are illustrated as points above and below the box-and-whisker diagram.

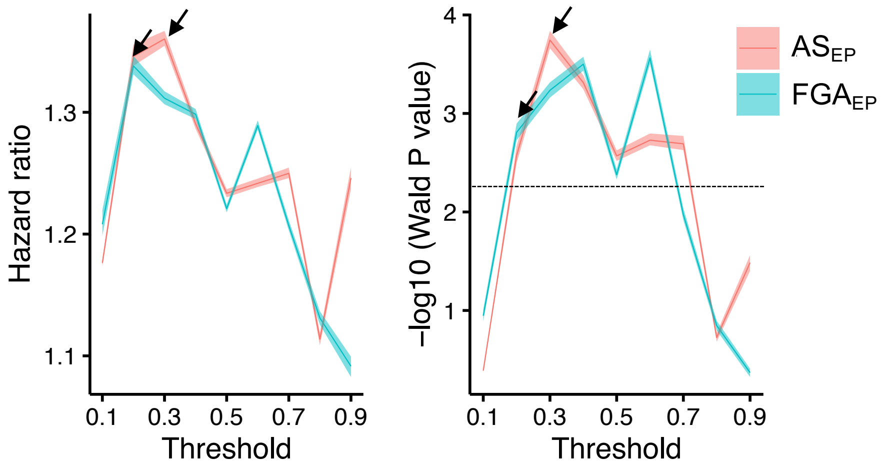

Supplementary Figure 5. Optimal binarization proportions ranging from 0.1 to 0.9 (corresponding to 10th through 90th percentiles) to split patients into high versus low (FGA or AS) scores.

Candidate binarization percentiles to split patients into high versus low FGA or AS scores calculated using cancer specific elbow point based CNA calling cutoff (FGA_{EP} and AS_{EP}) at each percentile. 1,660 multivariate Cox models as part of the leave-one-out cross validation analysis are constructed with FGA or AS (binned at the candidate binarization percentile), TMB (binned at the 80th percentile), and ICB drug class. The Wald p values and multivariate HRs with 95% confidence intervals are displayed respectively. Black arrows indicate Wald p-values and multivariable HRs at the optimal percentiles for FGA_{EP} (20th percentile) and AS_{EP} (30th percentile). Dashed line illustrates Bonferroni-corrected P = 0.05.

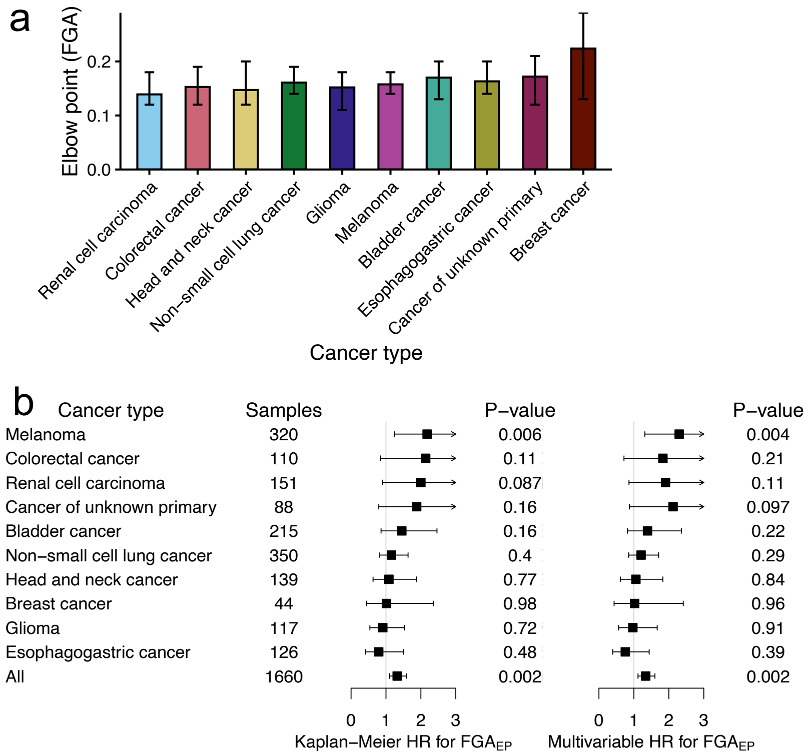

Supplementary Figure 6. An elbow-point based method to determine the cutoff used for calling CNAs.

(a) The elbow point values of the cutoff of |log2 copy ratio| in calculating FGA in individual cancer types. (**b**) Univariable Kaplan-Meier survival analysis and multivariable survival analysis using Cox proportional hazards regression of overall survival with FGA calculated using cancer specific elbow point based CNA calling cutoff (FGA_{EP}; binned at the 20th percentile), TMB (binned at the 80th percentile), and ICB drug class. Wald p values are displayed. In panel a, the bars represent 95% confidence intervals of the elbow point values calculated using a 1000-replicate bootstrapping. In panel b, squares positioned at midpoints symbolize point estimates of HRs, and the accompanying bars indicate 95% confidence intervals. The data are from the Samstein et al. cohort [1].

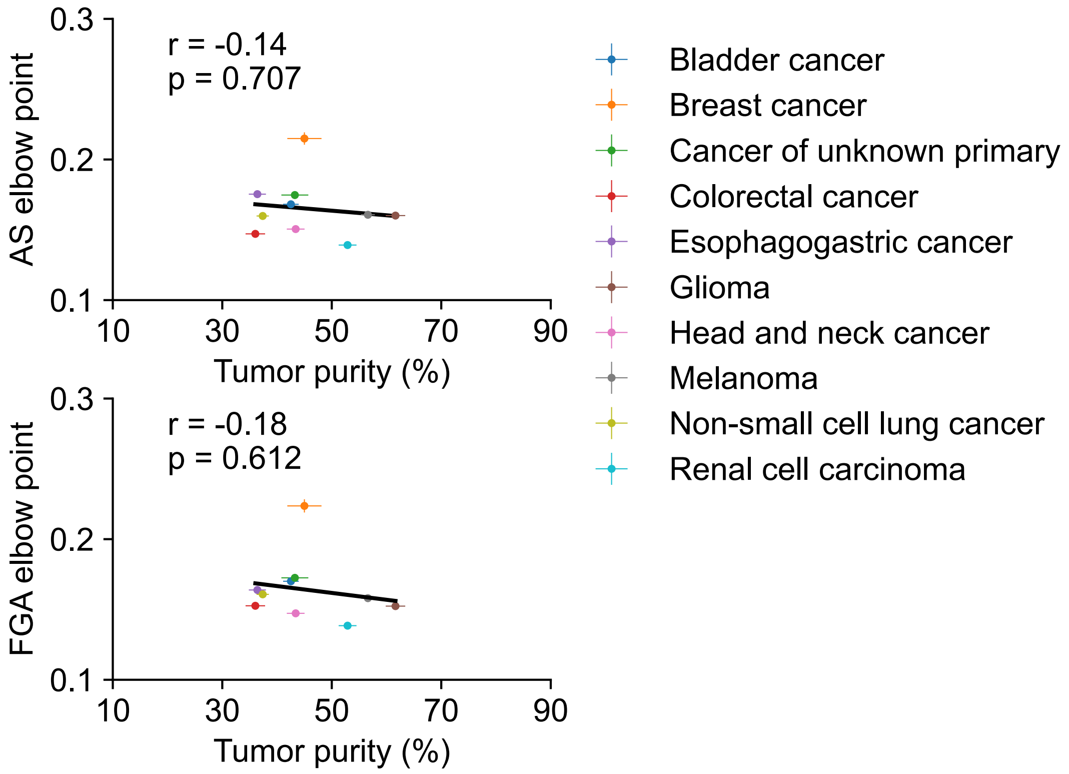

Supplementary Figure 7. Relationship between elbow points of CNA calling cutoff in calculating AS or FGA in individual cancer types and tumor purity.
Each data point in the figure represents the mean value of tumor purity and AS (or FGA) elbow point in a specific cancer type. The vertical bar on each point represents the standard error of the mean AS (or FGA) elbow point for patients within that cancer type. Similarly, the horizontal bar on each point represents the standard error of the mean tumor purity for patients within the corresponding cancer type.

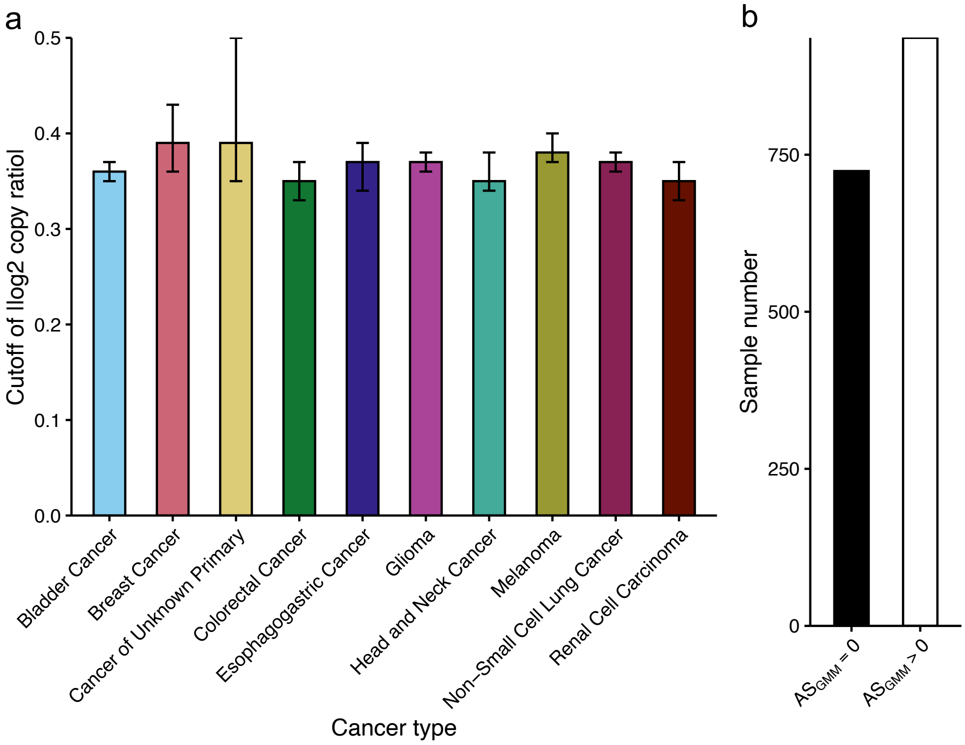

Supplementary Figure 8. The Gaussian mixture model (GMM) predicts unrealistically high CNA cutoff values.

(a) GMM-predicted CNA cutoffs for individual cancer types. **(b)** Number of samples with zero versus non-zero AS values calculated using GMM-predicted CNA cutoff values (AS_{GMM}); n = 1,660 biologically independent samples. In panel a, the bars represent 95% confidence intervals of the cutoff values calculated using a 1000-replicate bootstrapping.
