# Supplementary material for: Optimizing cancer immunotherapy response prediction by tumor aneuploidy score and fraction of copy number alterations
Source: NPJ Precis Oncol. 2023 Jun 3;7:54. doi: 10.1038/s41698-023-00408-6 (PMC10239491; doi:10.1038/s41698-023-00408-6)
Supplement: Supplementary file 2 — REPORTING SUMMARY [file 41698_2023_408_MOESM2_ESM.pdf]

nature portfolio

Corresponding author(s): Tiangen Chang, Eytan Ruppin

Last updated by author(s): Apr 20, 2023

Reporting Summary

Nature Portfolio wishes to improve the reproducibility of the work that we publish. This form provides structure for consistency and transparency in reporting. For further information on Nature Portfolio policies, see our Editorial Policies and the Editorial Policy Checklist.

Statistics

For all statistical analyses, confirm that the following items are present in the figure legend, table legend, main text, or Methods section.

n/a	Confirmed	
☐	☒	The exact sample size (*n*) for each experimental group/condition, given as a discrete number and unit of measurement
☒	☐	A statement on whether measurements were taken from distinct samples or whether the same sample was measured repeatedly
☐	☒	The statistical test(s) used AND whether they are one- or two-sided<br>*Only common tests should be described solely by name; describe more complex techniques in the Methods section.*
☐	☒	A description of all covariates tested
☐	☒	A description of any assumptions or corrections, such as tests of normality and adjustment for multiple comparisons
☐	☒	A full description of the statistical parameters including central tendency (e.g. means) or other basic estimates (e.g. regression coefficient) AND variation (e.g. standard deviation) or associated estimates of uncertainty (e.g. confidence intervals)
☐	☒	For null hypothesis testing, the test statistic (e.g. *F*, *t*, *r*) with confidence intervals, effect sizes, degrees of freedom and *P* value noted<br>*Give P values as exact values whenever suitable.*
☒	☐	For Bayesian analysis, information on the choice of priors and Markov chain Monte Carlo settings
☒	☐	For hierarchical and complex designs, identification of the appropriate level for tests and full reporting of outcomes
☐	☒	Estimates of effect sizes (e.g. Cohen's *d*, Pearson's *r*), indicating how they were calculated

Our web collection on statistics for biologists contains articles on many of the points above.

Software and code

Policy information about availability of computer code

Data collection	No software was used.
Data analysis	Data were used in the format downloaded from cBioPortal and the Supplementary Table of Chowell (et al 2022).<br>- Aneuploidy scores were called using ASCETS v1.1.1 (Spurr et al 2021).<br>- All statitics were performed in R v4.2 and Python 3.9.<br>- Kaplan–Meier survival analysis was performed using the R packages survminer v.0.4.9 and survival v.3.3.1.<br>- Elbow point method for determining CNA calling cutoff was calculated using Python package kneed v.0.8.1.<br>- Gaussian mixture model for determining CNA calling cutoff was calculated using Python package sklearn v.1.2.1.<br>- The power analysis of minimum sample size estimation for achieving statistically significant survival difference was performed using the R package powerSurvEpi v.0.1.3.<br>- Differential gene mutation frequency analysis was performed using the Python package scipy v.1.10.1.

For manuscripts utilizing custom algorithms or software that are central to the research but not yet described in published literature, software must be made available to editors and reviewers. We strongly encourage code deposition in a community repository (e.g. GitHub). See the Nature Portfolio guidelines for submitting code & software for further information.

Data

Policy information about availability of data

All manuscripts must include a data availability statement. This statement should provide the following information, where applicable:

- Accession codes, unique identifiers, or web links for publicly available datasets
- A description of any restrictions on data availability
- For clinical datasets or third party data, please ensure that the statement adheres to our policy

Data for the Samstein et al. cohort are available at https://www.cbioportal.org/study/summary?id=tmb_mskcc_2018 and the GENIE 20 v.7.1 release: https://www.synapse.org/#!Synapse:syn7222066/wiki/405659. Data for the Chowell et al. cohort are available from the supplementary table of 8, where FGA, TMB, ICB drug class, and overall survival information are provided. Aneuploidy scores were called using ASCETS at https://github.com/beroukhim-lab/ascets and values for each sample are provided in the following GitHub repository: https://github.com/rootchang/Aneuploidy-FGA-ICB.

Research involving human participants, their data, or biological material

Policy information about studies with human participants or human data. See also policy information about sex, gender (identity/presentation), and sexual orientation and race, ethnicity and racism.

Reporting on sex and gender	N/A
Reporting on race, ethnicity, or other socially relevant groupings	N/A
Population characteristics	N/A
Recruitment	N/A
Ethics oversight	N/A

Note that full information on the approval of the study protocol must also be provided in the manuscript.

Field-specific reporting

Please select the one below that is the best fit for your research. If you are not sure, read the appropriate sections before making your selection.

☒ Life sciences ☐ Behavioural & social sciences ☐ Ecological, evolutionary & environmental sciences

For a reference copy of the document with all sections, see nature.com/documents/nr-reporting-summary-flat.pdf

Life sciences study design

All studies must disclose on these points even when the disclosure is negative.

Sample size	All samples from the cBioPortal study listed above with copy-number segmentation files provided in GENIE v7.1 were included with the exception of one sample which was the sole non-melanoma skin cancer sample (n=1660). All samples from the Supplementary Table of Chowell (et al 2022) were included.
Data exclusions	No further samples were excluded except as described above.
Replication	Replication was performed for results from FGA0.2 on Chowell (et al 2022). No replication was performed for results from AS0.2 as the data were obtained from existing sources and no comparable datasets were available for validation.
Randomization	No radnomization was performed because this was a retrospective re-analysis of existing data.
Blinding	No blinding was performed because this was a retrospective re-analysis of existing data.

Reporting for specific materials, systems and methods

We require information from authors about some types of materials, experimental systems and methods used in many studies. Here, indicate whether each material, system or method listed is relevant to your study. If you are not sure if a list item applies to your research, read the appropriate section before selecting a response.

Materials & experimental systems

n/a	Involved in the study
☒	☐ Antibodies
☒	☐ Eukaryotic cell lines
☒	☐ Palaeontology and archaeology
☒	☐ Animals and other organisms
☒	☐ Clinical data
☒	☐ Dual use research of concern
☒	☐ Plants

Methods

n/a	Involved in the study
☒	☐ ChIP-seq
☒	☐ Flow cytometry
☒	☐ MRI-based neuroimaging
