# Supplementary material for: Optimizing cancer immunotherapy response prediction by tumor aneuploidy score and fraction of copy number alterations
Source: NPJ Precis Oncol. 2023 Jun 3;7:54. doi: 10.1038/s41698-023-00408-6 (PMC10239491; doi:10.1038/s41698-023-00408-6)
Supplement: Supplementary file 3 — CC [file 41698_2023_408_MOESM3_ESM.pdf]

tiangen.pdf

WORD COUNT 7476

TIME SUBMITTED 23-MAY-2023 01:32PM

PAPER ID 99913664

Optimizing cancer immunotherapy response prediction by tumor aneuploidy score and fraction of copy number alterations

Tiangen Chang[1,$], Yingying Cao[1], Eldad D. Shulman[1], Uri Ben-David[2], Alejandro A. Schäffer[1], Eytan Ruppin[1,$]

[1] Cancer Data Science Laboratory, Center for Cancer Research, National Cancer Institute (NCI), National Institutes of Health (NIH), Bethesda, MD, USA 20892

[2] Department of Human Molecular Genetics and Biochemistry, Faculty of Medicine, Tel Aviv University, Tel Aviv, Israel

$ Corresponding authors

Emails for corresponding authors: tiangen.chang@nih.gov; eytan.ruppin@nih.gov.

ABSTRACT

Identifying patients that are likely to respond to cancer immunotherapy is an important, yet highly challenging clinical need. Using 3,139 patients across 17 different cancer types, we comprehensively studied the ability of two common copy number alteration (CNA) scores – the tumor aneuploidy score (AS) and the fraction of genome single nucleotide polymorphism encompassed by copy number alterations (FGA) – to predict survival following immunotherapy in both pan-cancer and individual cancer types. Firstly, we show that choice of cutoff during CNA calling significantly influences the predictive power of AS and FGA for patient survival following immunotherapy. Remarkably, by using proper cutoff during CNA calling, AS and FGA can predict pan-cancer survival following immunotherapy for both high TMB and low TMB patients. However, at individual cancer level, our data suggests that the use of AS and FGA for predicting immunotherapy response is currently limited to only a few cancer types. Therefore, larger sample sizes are needed to evaluate the clinical utility of these measures for patient stratification in other cancer types. Finally, we propose a simple, non-parameterized, elbow-point based method to help determine the cutoff used for calling CNAs.

INTRODUCTION

Although various studies have shown that high tumor mutation burden (TMB) may predict immunotherapy response, at least in some cancer types [1,2], more precise identification of patients that are likely to respond to cancer immunotherapy is still a challenging unmet clinical need. One promising approach to identify responders of immunotherapy has been to study the predictive ability of other measures of genomic alterations in cancer in these patients. Two natural candidates are scores based on copy number alterations (CNAs): (a) tumor aneuploidy, which measures chromosome-level CNAs, and (b) global genomic CNAs, which quantifies the extents of both chromosomal and focal copy-number events [3]. Both tumor aneuploidy and genomic CNAs have been shown to play a role in cancer progression and to be predictive for cancer prognosis [3-5].

Recently, Spurr et al. reported that the tumor *aneuploidy score* (AS), defined as the fraction of chromosome arms with arm-level CNAs in a sample, which was called using a loose cutoff of $|\log 2 \text{ copy ratio}| > 0.1$, is significantly predictive of survival following immunotherapy in low TMB patients, but not in high TMB patients, in a pan-cancer analysis [6]. In addition, they reported that AS had stronger predictive power than another metric conceptually related to the AS, the *fraction of genome encompassed by copy number alterations* (FGA) which quantifies the extent of both chromosomal and focal copy-number events [6]. As FGA combines both chromosomal and focal CNAs, if the association between CNAs and immunotherapy response is driven by the overall genomic instability, one would expect FGA to perform at least as well as AS in predicting immunotherapy response. Therefore, the conclusion of [6] that AS is a better predictor than FGA in low TMB patients is non-intuitive. Intrigued by these potentially clinically

impactful findings, we set out to explore several related fundamental questions: (1) Does the choice of cutoff during CNA calling influence the predictive power? (2) Are AS and FGA also predictive of survival for high TMB patients? (3) Are AS and FGA predictive of survival of patients following immunotherapy in *individual* cancer types?

RESULTS

The Choice of cutoff during CNA calling markedly influences the predictive power of AS and FGA for patient survival following immunotherapy

We first re-analyzed the same data used by Spurr et al. [6], i.e., the Samstein et al. cohort [1] from MSK-IMPACT (data acquired from the AACR Project GENIE). This study analyzed a published cohort of 1,660 advanced cancer patients from 10 different cancer types treated with immune checkpoint blockade (ICB). Their results show that, at the pan-cancer level, a higher AS was associated with worse survival following immunotherapy among patients with low TMB (defined as the bottom 80% of TMB in each cancer type). However, their study did not explicitly identify the individual cancer types in which AS is predictive. As a pan-cancer Kaplan-Meier survival analysis (as performed by Spurr et al. [6]) may be confounded by the cancer-type composition of the overall dataset, and as most clinical trials usually focus on individual cancer types, we first set out to compare the Kaplan-Meier survival curves of low TMB patients with high versus low AS for each of the 10 cancer types individually.

The initial cancer-type-specific analysis was performed by using the AS values provided by [6] (which calls chromosome-level CNAs using the cutoff of |log2 copy ratio| > 0.1; denoted as $AS_{0.1}$). Unexpectedly, a Kaplan-Meier survival analysis of low TMB patients identified a

statistically significant worse survival following immunotherapy in a single individual cancer type, i.e., "cancer of unknown primary", which refers to a group of cancers with unknown origin, often due to metastasis making it difficult to locate the primary site (n = 70, hazard ratio HR = 2.27, p = 0.031; Supplementary Fig. 1). Here, the HR denotes the relative risk of the $AS_{0.1}$-high individuals compared to the $AS_{0.1}$-low set as the reference.

Aiming to improve on these results, we observed that while the cutoff used to determine a CNA event in [6] was |log2 copy ratio| > 0.1, the cutoff of |log2 copy ratio| > 0.2 in calculating AS and/or FGA was more frequently used (e.g. [7-9]). Our first hypothesis tested whether the choice of cutoff during CNA calling affects the predictive power of AS and FGA for survival following immunotherapy. To this end, we re-calculated AS and FGA for each sample using the CNA calling cutoff of |log2 copy ratio| > 0.2. We then compared the HRs of AS and FGA in individual cancer types using AS and FGA respectively in a multivariable Cox proportional-hazards regression of overall survival with TMB and ICB drug class, as had been done in [6]. Remarkably, HRs were significantly increased for both AS (p = 0.019) and FGA (p = 0.032) by using the CNA calling cutoff of |log2 copy ratio| > 0.2 (denoted by $AS_{0.2}$ and $FGA_{0.2}$ respectively) compared to that calculated by using cutoff of 0.1 in [6] (denoted by $AS_{0.1}$ and $FGA_{0.1}$ respectively; Fig. 1).

AS and FGA predict pan-cancer survival following immunotherapy for both high TMB and low TMB patients

Although $AS_{0.2}$ and $FGA_{0.2}$ are continuous variables, a binary score (based on high or low scores) is preferred in clinical decision making. Following [6], we determined the percentile to

partition the $AS_{0.2}$ scores into low and high so that they are optimally synergized with TMB to risk-stratify patients following immunotherapy by testing every tenth quantile within each cancer type, moving in increments from the 20th to 80th percentile, using a multivariate model with TMB (binned at 80th percentile) and ICB drug class. We identified the 60th percentile in each cancer type as the optimal binarization threshold to classify patients into high $AS_{0.2}$ and low $AS_{0.2}$ groups because it yielded highest multivariate HR with significant Bonferroni-corrected p-value (Fig. 2a). Similarly, the optimal percentiles to binarize the $AS_{0.1}$, $FGA_{0.1}$, and $FGA_{0.2}$ scores are 50th, 40th, and 50th, respectively (Fig. 2a).

Then, we tested our second hypothesis, examining whether $AS_{0.2}$ and $FGA_{0.2}$ can predict survival outcomes for both high TMB patients and low TMB patients. Strikingly, both AS0.2 and FGA0.2 had similar effect size in predicting survival in high TMB patients compared with that in low TMB patients, respectively. Specifically, the HRs between high and low $AS_{0.2}$ groups were 1.23 and 1.34, respectively, among high TMB versus low TMB patients (Fig. 2b); Similarly, the HRs between high and low $FGA_{0.2}$ groups were 1.32 and 1.35, respectively, among high TMB versus low TMB patients (Fig. 2c). Overall, by using TMB and $AS_{0.2}$ (or $FGA_{0.2}$) together, we can classify patient survival following immunotherapy into four groups: high TMB & low $AS_{0.2}$ (or $FGA_{0.2}$) > high TMB & high $AS_{0.2}$ (or $FGA_{0.2}$) > low TMB & low $AS_{0.2}$ (or $FGA_{0.2}$) > low TMB & high $AS_{0.2}$ (or $FGA_{0.2}$). Additionally, $FGA_{0.2}$ was found to have consistently slightly higher HRs and lower p-values than $AS_{0.2}$ (Fig. 2a-c), which suggests that $FGA_{0.2}$ is better or performs at least as well as $AS_{0.2}$ in predicting pan-cancer ICB response.

To further test this finding in other datasets, we analyzed another MSK-IMPACT cohort published recently by Chowell et al. [8] (data from the supplementary table of the paper). In the Chowell et al. cohort, there are in total 15 cancer types, 8 of them are in common with the above used Samstein et al. cohort (we merged gastric and esophageal cancers in the Chowell et al. cohort into esophagogastric cancer to keep in line with the tumor type classification in the Samstein et al. cohort). We note that we could not use the Chowell et al. data to validate the AS analysis because these data do not include AS values and it is not possible to calculate the AS values based on the publicly available information. Consistently, $FGA_{0.2}$ was found to predict survival following immunotherapy for both high TMB and low TMB patients. Specifically, the HRs between high and low $FGA_{0.2}$ groups were 1.63 and 1.18, respectively, among high TMB versus low TMB patients (Fig. 2d).

We hypothesized that CNA calling cutoff |log2 copy ratio| > 0.1 is a too low cutoff, which introduced noise in calculating patient AS, and thus dampened its predictive power of survival following immunotherapy. To test this hypothesis, we divided patients in the Samstein et al. cohort into four groups by their high / low $AS_{0.1}$ / $AS_{0.2}$ scores and compared the Kaplan-Meier survival curves (Fig. 2e). We found that, among high $AS_{0.2}$ or among low $AS_{0.2}$ patients, there was no significant survival difference between patients that had high or low $AS_{0.1}$ values. In contrast, among high $AS_{0.1}$ patients, a subset of patients, i.e., the low $AS_{0.2}$ patients, had much better survival rates than high $AS_{0.2}$ patients (HR = 1/1.33 = 0.75, p-value = 0.009); they actually achieved similar survival rates as the low $AS_{0.1}$ / low $AS_{0.2}$ patients (HR = 1, p-value = 0.9). On the other hand, among low $AS_{0.1}$ patients, a subset of patients, i.e., the high $AS_{0.2}$ patients, had significantly worse survival rates than low $AS_{0.2}$ patients (HR = 1.34, p-value = 0.06); they

actually had similar survival rates as the high $AS_{0.1}$ / high $AS_{0.2}$ patients (HR = 1, p-value = 0.9). This result testifies that the $AS_{0.1}$ indeed mis-classifies a number of patients as a result of the loose CNA calling cutoff used. Further investigation into the patients that were misclassified by $AS_{0.1}$ showed that the "low $AS_{0.2}$, high $AS_{0.1}$" patients had significantly lower tumor purity than the "high $AS_{0.2}$, high $AS_{0.1}$" patients; and similarly, the "high $AS_{0.2}$, low $AS_{0.1}$" patients had significantly higher tumor purity than the "low $AS_{0.2}$, low $AS_{0.1}$" patients (Supplementary Fig. 2). These findings suggest that tumor purity may, at least partially, explain the switch of some samples from high/low $AS_{0.1}$ to low/high $AS_{0.2}$. However, further studies are needed to fully understand the relationship between AS and tumor purity and to determine the optimal cutoff for AS in predicting patient response to immunotherapy when such data are available.

Tumor aneuploidy score ($AS_{0.2}$) and fraction of copy number alterations ($FGA_{0.2}$) predict survival following immunotherapy in certain individual cancers

Having demonstrated that $AS_{0.2}$ and $FGA_{0.2}$ predict survival following immunotherapy for both high TMB and low TMB patients at the pan-cancer level, we next asked whether these scores could also predict survival in *individual* cancer types. As a result, in the Samstein et al. cohort, $FGA_{0.2}$ had significant HRs for pan-cancer (HR = 1.36, $p < 0.0001$) and in three individual cancer types in Kaplan-Meier survival analysis, i.e., renal cell carcinoma (HR = 2.03, $p = 0.01$), melanoma (HR = 1.78, $p = 0.002$), and bladder cancer (HR = 1.73, $p = 0.009$; Fig. 3a). In comparison with $FGA_{0.2}$, AS0.2, $AS_{0.2}$ yielded significant Kaplan-Meier univariable HRs in bladder cancer and renal cell carcinoma, and marginally significant multivariate HRs in melanoma (Supplementary Fig. 3). Comparison of HRs using $AS_{0.2}$ or $FGA_{0.2}$ in a multivariable Cox model with TMB (binned at the 80th percentile) and ICB drug class yielded very similar

result (Fig. 3a; Supplementary Fig. 3). Overall, we conclude that FGA performs comparable to or better than AS in predicting immunotherapy response in individual cancers, suggesting that it is the overall genome affected by CNAs (rather than the individual CNA length or mechanism of formation) that drives the observed CNA-immunotherapy response associations.

We further tested the robustness of $FGA_{0.2}$ in predicting survival following immunotherapy in the other dataset, i.e., the Chowell et al. cohort. As a result, $FGA_{0.2}$ had significant Kaplan-Meier univariable HRs for pan-cancer (HR = 1.22, p = 0.003) and in renal cell carcinoma (HR = 2.07, p = 0.019) and melanoma (HR = 1.75, p = 0.018). Again, multivariable Cox model with adjustment of TMB (binned at the 80th percentile) and ICB drug class yielded similar result (Fig. 3b). However, $FGA_{0.2}$ did not predict worse survival for high $FGA_{0.2}$ in bladder cancer in this cohort as what in the Samstein et al. cohort (HR = 0.79, p = 0.39; Fig. 3b), which might be due to the small sample size in the Chowell et al. cohort (n = 82; Fig. 3b), and / or, due to tumor heterogeneity. For example, further comparison analysis showed that bladder cancer samples in the Samstein et al. cohort had slightly lower mean $FGA_{0.2}$ value (0.19 versus 0.23, p = 0.14) and better survival (HR = 0.75, p = 0.098) than that in the Chowell et al. cohort (Supplementary Fig. 4).

Interestingly, $FGA_{0.2}$ predicted significant HRs for one more cancer type, non-small cell lung cancer, although with modest HR values (HR = 1.25, p = 0.05; Fig. 3b). Given that the HR values in the Samstein et al. cohort show a similar trend but are non-significant (HR = 1.19, p = 0.19; Fig. 3a), we wondered whether the difference in statistical significance might be due to the difference of sample size. Therefore, we performed a power analysis to estimate the sample size

needed for achieving statistical significance p value less than 0.05. The estimated sample size for non-small cell lung cancer in the Samstein et al. cohort to achieve $p < 0.05$ is about 1600 (Supplementary Table 1). Similarly, it was found that colorectal cancer might also achieve significant HR > 1 with a sample size of ~600 patients in both cohorts (Supplementary Table 1). Our data analysis revealed that significant Kaplan-Meier survival analysis always corresponded to significant multivariate analysis in individual cancer types and vice versa. Therefore, this is unlikely the reason that AS/FGA in certain cancer types, and vice versa. Therefore, it is unlikely that the limited efficacy of AS/FGA in certain cancer types is due to differences in statistical significance. Instead, it is more plausible that AS/FGA may not work in certain cancer types due to specific underlying biological factors. As previously discussed, sample size limitation may also be a critical factor for most specific cancer types, as suggested by our power analysis.

In addition, to investigate whether mutation of specific genes may contribute to the survival difference following immunotherapy, we performed a differential gene mutation frequency analysis among high $FGA_{0.2}$ (or $AS_{0.2}$) group versus low $FGA_{0.2}$ (or $AS_{0.2}$) group in the two cancer types in the Samstein et al. cohort with the largest sample size, melanoma and non-small cell lung cancer. We found that none of the genes had significantly differential mutation frequencies between the high $FGA_{0.2}$ (or $AS_{0.2}$) versus low $FGA_{0.2}$ (or $AS_{0.2}$) patients after multiple testing correction (Supplementary Table 2).

The elbow-point based method offers one systematic way to determine the cutoff used for calling CNAs

Finally, as shown above, the cutoff used for calling CNAs is critical for calculating $AS_{0.2}$ and $FGA_{0.2}$. A low cutoff of |log2 copy ratio| in calling CNA events might introduce noise (false positives), whereas a high cutoff might result in missing true events (false negatives). There are a number of parameters that may affect the optimal cutoff, e.g., cancer type, tumor purity, and the platform used for CNA calling (e.g., whole exome sequencing, single nucleotide polymorphism arrays, and shallow whole genome sequencing) [10-14]. The variance of these parameters in different cancer types is likely to explain why AS and FGA scores have very different predictive power in distinct cancer types. We hence reasoned that an arbitrary threshold could never be optimal for all datasets and searched for an unbiased approach for threshold calling. We used the "elbow method", which was developed to identify a cutoff point that optimally distinguishes between two qualitative, discrete states [15]. This method has been found to be effective in determining optimal parameter thresholds in a variety of data-driven optimization tasks including the determination of the number of clusters, determination of the number of principal components, and with relevance to our goal, determination of the threshold on a receiver operating characteristic (ROC) curve [16-18].

We calculated the elbow points of CNA calling cutoff |log2 copy ratio| for AS for all 10 individual cancer types (exemplified as in Fig. 4a), which are in the range of 0.14-0.22 with 95% confidence interval (CI) in the range of 0.12-0.27 (Fig. 4b). Therefore, the cutoff of 0.1 used in [6] is well-below the elbow points for all individual cancer types. However, on the other hand, the average values of elbow points across different cancer types of both AS and FGA are 0.17, which is very close to the cutoff of 0.2 used above. These facts may explain why the 0.2 cutoff performs much better than the 0.1 cutoff. We further re-evaluated the predictive power of AS by

calculating AS using the elbow points as the CNA calling cutoff per cancer types (denoted as AS_{EP}). We identified the 30th percentile as the optimal binarization threshold to classify patients into high AS_{EP} and low AS_{EP} groups (Supplementary Fig. 5). The multivariable HRs of binarized AS_{EP} (with adjustment of TMB and ICB drug class) in individual cancer types were, on average, greater than those obtained using $AS_{0.1}$ (Δ mean HR = 0.21, p = 0.08; Fig. 4c). Furthermore, AS_{EP} predicted significant HR in melanoma and marginally significant HRs in two other cancer types, i.e., non-small cell lung cancer and renal cell carcinoma, tested by both Kaplan-Meier univariable survival analysis and multivariable Cox model with adjustment for TMB and ICB drug (Fig. 4c,d). The elbow-point based method to determine the cutoff used for calling CNAs yielded similar result in FGA (Supplementary Figs. 5, 6). To test if differential tumor purity across different cancer types may contribute to the variation of elbow points in individual cancer types, we investigated the relationship between elbow points and average tumor purity. A weak but statistically nonsignificant negative correlation was found (Supplementary Fig. 7).

In addition, we also tested using another method to determine the cutoff, testing a Gaussian mixture model (GMM). However, the Gaussian mixture model gave unrealistic high cutoff values ranging from 0.35-0.39 for individual cancer types (Supplementary Fig. 8a), which resulted in AS=0 for nearly half of the samples (Supplementary Fig. 8b).

Taken together, these results suggest that the elbow method, a simple and non-parametric method, is robust and superior to some arbitrarily chosen cutoffs (e.g. the 0.1 cutoff used by [6]). However, it was not possible to further test the elbow method in the Chowell et al. cohort due to the inaccessibility of some of the data. In the future, the elbow-point based method needs to be

tested in more cohorts to further validate it. Moreover, as tumor purity and ploidy information of samples per tumor type are important factors in detecting CNAs, more sophisticated methods (e.g., iChorCNA[12], Accurity[14]) are needed to take this information into consideration before determining the cutoff for CNA calling when such data are available.

DISCUSSION

In summary, we have comparatively assessed the power of AS and FGA in predicting patient survival following immunotherapy in pan-cancer and individual cancer types. Addressing our research questions, we first show that choice of cutoff during CNA calling greatly influences the predictive power of AS and FGA for patient survival following immunotherapy. Specifically, the AS measure defined in [6] ($AS_{0.1}$) hardly significantly predict survival benefit following immunotherapy in low TMB patients in any single cancer type (Supplementary Fig. 1). $AS_{0.2}$ and $FGA_{0.2}$, re-calculated using a more appropriate pan-cancer CNA calling cutoff of $|\log2 \text{ copy ratio}| > 0.2$, have considerably stronger predictive power of survival following immunotherapy (Fig. 1). Secondly, we show that $AS_{0.2}$ and $FGA_{0.2}$ predict pan-cancer survival following immunotherapy for both high TMB and low TMB patients, rather than in low TMB patients only, as was claimed in [6]; as evidence, the arbitrary cutoff of $|\log2 \text{ copy ratio}| > 0.1$ used in [6] is found to misclassify many patients (Fig. 2). Finally, from a translational standpoint, the currently available data suggest that both AS and FGA can only significantly predict survival following immunotherapy in a few cancer types (Figs. 3, 4). Therefore, larger sample sizes are required to evaluate, and ultimately use these measures within individual cancer types.

METHODS

Patient samples

Data for the Samstein et al. [1] cohort (MSK-IMPACT) were downloaded from cBioPortal at http://www.cbioportal.org/study?id= tmb_mskcc_2018. Segmented copy-number data were downloaded from AACR Project GENIE v.7.1. Note that one sample of "skin cancer - nonmelanoma" was excluded from the analyses as it was the only sample representing this specific histology. Data for the Chowell et al. cohort were obtained from the supplementary table of [8].

Copy number alteration and tumor aneuploidy assessment

AS were calculated using ASCETS v.1.1 at https://github.com/beroukhim-lab/ascets with the following command line:

script run_ascets.R -i genie_msk_cna_hg19.seg -c genomic_arm_coordinates_hg19.txt -o ./output/output*x* -t *x*

where x is the CNA calling cutoff, defined as the threshold at which a CNA event is counted if |log2 copy ratio| > x.

FGA was calculated as the ratio between the sum of the lengths of the genomic segments with |log2 copy ratio| > x, and the sum of the lengths of all measured segments:

FGA = sum(seg_length[abs(seg.mean) >= *x*]) / sum(seg_length)

CNA calling cutoff point determination

The elbow-point based method: CNA events, which were used to calculate AS and FGA, were firstly called using |log2 copy ratio| cutoffs ranging from 0.01 to 0.5 with a step size of 0.01.

Then, to calculate the cancer-type-specific elbow points of cutoffs, mean values of AS/FGA across samples in individual cancer types were calculated under each cutoff to generate the AS/FGA-cutoff curves. Finally, the cancer-type-specific elbow point in each bootstrap replication was calculated using Python package *kneed* v.0.8.1; and 95% confidence intervals of elbow points were determined from 1000-replicate bootstrapping.

The Gaussian mixture model: |log2 copy ratio| cutoffs were calculated in a similar way as the elbow method, except that in the final step, the cutoff point was determined by the Gaussian mixture model with two components using the *GaussianMixture*() function in the Python package *sklearn* v.1.2.1. This model assumes that the data is generated from two Gaussian distributions with different means and variances, and that each data point belongs to one of the two distributions with a certain probability.

Binarization of TMB, AS, and FGA

The patient TMB, AS, and FGA values were binarized into score-high versus score-low groups in a cancer-type-specific manner. Specifically, in each cancer type, the patients who had the top 20% of the TMB values were classified into high TMB group, while others were classified into low TMB group following [1]. To determine the optimal binarization of AS and FGA that effectively synergized with TMB for risk stratification of patients undergoing immunotherapy, we performed a comprehensive analysis. This involved testing every tenth quantile within each cancer type, ranging from the 20th to 80th percentile, using a multivariate model that incorporated TMB (binned at the 80th percentile) and ICB drug class following [6]. Leave-one-out cross-validation was conducted to identify the optimal threshold for defining high versus low AS

(or FGA). In detail, for each threshold, we constructed a Cox proportional hazards survival model incorporating binarized AS (or FGA), TMB, and drug class. This process was repeated iteratively for the cohort size (1,660), with one unique patient left out in each iteration. The goal was to identify the threshold that yielded the highest multivariate hazard ratio (HR) in synergy with TMB, while maintaining a significant Bonferroni-corrected p-value (see **Fig. 2a**).

Statistical analysis

Survival analysis: Kaplan–Meier survival analysis was performed using the R packages *survminer* v.0.4.9 and *survival* v.3.3.1, and HR and P values were calculated with univariable Cox proportional hazard regression using the *coxph*() function [19]. Multivariable analysis was performed with Cox proportional hazard regression in individual cancer types, with inclusion of covariates including FGA (or AS), TMB and ICB drug class.

Power analysis: The power analysis of minimum sample size estimation for achieving statistically significant survival difference (Kaplan-Meier HR > 1, $p < 0.05$) in individual cancer types in the Samstein et al. cohort was performed using the R package *powerSurvEpi* v.0.1.3 with parameter "power = 0.8", which means that there is an 80% chance of correctly detecting a statistically significant effect if one exists.

Gene mutation frequency analysis: We defined the gene mutation frequency in a group of patients as the fraction of patients with mutations in the gene of interest. To identify genes with significantly different mutation frequencies between AS (or FGA) high and low groups, we compared the gene mutation frequencies in the two groups using the chi-squared test. We used

the *chi2_contingency*() function from the Python package *scipy* v.1.10.1 to perform the chi-squared test. To correct for multiple testing, we applied the Bonferroni correction.

DATA AVAILABILITY

Data for the Samstein et al. cohort are available at https://www.cbioportal.org/study/summary?id=tmb_mskcc_2018 and the GENIE [20] v.7.1 release: https://www.synapse.org/#!Synapse:syn7222066/wiki/405659. Data for the Chowell et al. cohort are available from the supplementary table of [8] at https://static-content.springer.com/esm/art%3A10.1038%2Fs41587-021-01070-8/MediaObjects/41587_2021_1070_MOESM3_ESM.xlsx, where FGA, TMB, ICB drug class, and overall survival information are provided. Aneuploidy scores were called using ASCETS at https://github.com/beroukhim-lab/ascets and values for each sample are provided in the GitHub repository at https://github.com/rootchang/Aneuploidy-FGA-ICB.

CODE AVAILABILITY

All code necessary to replicate these analyses is provided in the following GitHub repository: https://github.com/rootchang/Aneuploidy-FGA-ICB.

ACKNOWLEDGMENTS

This research was supported in part by the NIH Intramural Research Program, National Cancer Institute. This work utilized the computational resources of the NIH HPC Biowulf cluster (http://hpc.nih.gov). The authors would like to acknowledge the American Association for

Cancer Research and its financial and material support in the development of the AACR Project GENIE registry, as well as members of the consortium for their commitment to data sharing. Interpretations are the responsibility of study authors.

AUTHOR CONTRIBUTIONS

T.C. and E.R. conceived and designed the study. T.C. and Y.C. collected and managed the data. T.C., Y.C. and E.D.S. performed the statistical analyses. U.B.-D. and A.A.S. provided statistical advice. All authors critically revised the manuscript for important intellectual content.

COMPETING INTERESTS

E.R. is a co-founder of MedAware, Metabomed and Pangea Biomed (divested), and an unpaid member of Pangea Biomed's scientific advisory board. U.B.-D. receives grant funding from Novocure. The other authors have no competing interests.

REFERENCES

1. Samstein, R.M. *et al.* Tumor mutational load predicts survival after immunotherapy across multiple cancer types. *Nat. Genet.* **51**, 202-206 (2019).
2. McGrail, D.J. *et al.* High tumor mutation burden fails to predict immune checkpoint blockade response across all cancer types. *Ann. Oncol.* **32**, 661-672 (2021).
3. Ben-David, U. & Amon, A. Context is everything: aneuploidy in cancer. *Nat. Rev. Genet.* **21**, 44-62 (2020).
4. Hieronymus, H. *et al.* Tumor copy number alteration burden is a pan-cancer prognostic factor associated with recurrence and death. *Elife* **7** https://doi.org/10.7554/eLife.37294 (2018).
5. Sansregret, L. & Swanton, C. The Role of Aneuploidy in Cancer Evolution. *Cold Spring Harbor Perspectives in Medicine* **7** https://doi.org/10.1101/cshperspect.a028373 (2017).
6. Spurr, L.F., Weichselbaum, R.R. & Pitroda, S.P. Tumor aneuploidy predicts survival following immunotherapy across multiple cancers. *Nat. Genet.* **54**, 1782-1785 (2022).
7. Spurr, L.F. *et al*. Quantification of aneuploidy in targeted sequencing data using ASCETS. *Bioinformatics* **37**, 2461-2463 (2021).
8. Chowell, D. *et al.* Improved prediction of immune checkpoint blockade efficacy across multiple cancer types. *Nat. Biotechnol.* **40**, 499-506 (2022).
9. Rizvi, H. *et al.* Molecular determinants of response to anti–programmed cell death (PD)-1 and anti–programmed death-ligand 1 (PD-L1) blockade in patients with non–small-cell lung cancer profiled with targeted next-generation sequencing. *J. Clin. Oncol.* **36**, 633-641 (2018).

10. Woo, X.Y. *et al.* Conservation of copy number profiles during engraftment and passaging of patient-derived cancer xenografts (vol 53, pg 86, 2021). *Nat. Genet.* **53**, 761-761 (2021).

11. Beroukhim, R. *et al.* The landscape of somatic copy-number alteration across human cancers. *Nature* **463**, 899-905 (2010).

12. Adalsteinsson, V.A. *et al.* Scalable whole-exome sequencing of cell-free DNA reveals high concordance with metastatic tumors. *Nat. Commun.* **8** https://doi.org/10.1038/s41467-017-00965-y (2017).

13. Hoge, A.C.H. *et al.* DNA-based copy number analysis confirms genomic evolution of PDX models. *npj Precis. Onc.* **6** https://doi.org/10.1038/s41698-022-00268-6 (2022).

14. Luo, Z.H., Fan, X.P., Su, Y. & Huang, Y.S. Accurity: accurate tumor purity and ploidy inference from tumor-normal WGS data by jointly modelling somatic copy number alterations and heterozygous germline single-nucleotide-variants. *Bioinformatics* **34**, 2004-2011 (2018).

15. Satopaa, V., Albrecht, J., Irwin, D. & Raghavan, B. Finding a "kneedle" in a haystack: Detecting knee points in system behavior. *2011 31st International Conference on Distributed Computing Systems Workshops* 166-171 (IEEE, 2011).

16. Syakur, M.A., Khotimah, B.K., Rochman, E.M.S. & Satoto, B.D. Integration k-means clustering method and elbow method for identification of the best customer profile cluster. *IOP Conf. Ser.: Mater. Sci. Eng.* **336**, 012017 (2018).

17. Linting, M., Meulman, J.J., Groenen, P.J.F. & van der Kooij, A.J. Nonlinear principal components analysis: Introduction and application. *Psychol. Methods* **12**, 336-358 (2007).

18. Oh, J.H., Hong, J.Y. & Baek, J.G. Oversampling method using outlier detectable generative adversarial network. *Expert Syst. Appl.* **133**, 1-8 (2019).

19. Therneau, T. A package for survival analysis in S. *R package version* **2**(7) (2015).

20. Andre, F. *et al.* AACR Project GENIE: Powering Precision Medicine through an International Consortium. *Cancer Discov.* **7**, 818-831 (2017).

FIGURE LEGENDS

Figure 1. The choice of cutoff during CNA calling markedly influences the predictive power of AS and FGA for patient survival following immunotherapy.

Comparison of HRs using $AS_{0.1}$ or $AS_{0.2}$ or $FGA_{0.1}$ or $FGA_{0.2}$ in a multivariate Cox model with TMB and ICB drug class. Paired Wilcoxon test p values are displayed. In the plot, the upper and lower boundaries signify the first and third quartiles, correspondingly, the central line denotes the median, and the whiskers stretch to the most distant data points not classified as outliers (within 1.5 times the interquartile range). The data are from the Samstein et al. cohort [1].

Figure 2. AS and FGA predict pan-cancer survival following immunotherapy for both high TMB and low TMB patients.

(a) The x-axis shows candidate binarization proportions 0.1 through 0.9 corresponding to 10th through 90th percentiles to partition patient scores into high score versus low score at each percentile. 1,660 multivariate Cox models as part of the leave-one-out cross validation analysis are constructed with $AS_{0.1}$ or $AS_{0.2}$ or $FGA_{0.1}$ or $FGA_{0.2}$ (binned at the candidate binarization percentile), TMB (binned at the 80th percentile), and ICB drug class. The Wald p values and multivariate HRs with 95% confidence intervals are displayed respectively. Black arrows indicate Wald p-values and multivariable HRs at the optimal percentiles respectively. Dashed line denotes the Bonferroni-corrected P = 0.05. **(b)** Pan-cancer Kaplan-Meier analysis of $AS_{0.2}$ binned at the 60th percentile and TMB binned at the 80th percentile in the Samstein et al. cohort. **(c-d)** Pan-cancer Kaplan-Meier analysis of $FGA_{0.2}$ binned at the 50th percentile and TMB binned at the 80th percentile in the Samstein et al. cohort (c) and in the Chowell et al. cohort (d). **(e)** Pan-cancer Kaplan-Meier analysis of $AS_{0.2}$ binned at the 60th percentile and $AS_{0.1}$ binned at the

50th percentile in the Samstein et al. cohort. HR and p values of pairwise comparisons between different groups are shown. H, high; L, low.

Figure 3. Fraction of copy number alterations ($FGA_{0.2}$) predict survival following immunotherapy in certain individual cancers.

Univariable Kaplan-Meier survival analysis and multivariable survival analysis using Cox proportional hazards regression of overall survival with $FGA_{0.2}$ (binned at the 50th percentile), TMB (binned at the 80th percentile), and ICB drug class in the Samstein et al. cohort (**a**) and in the Chowell et al. cohort (**b**). In the plot, squares positioned at midpoints symbolize point estimates of HRs, and the accompanying bars indicate 95% confidence intervals. Wald p values are displayed.

Figure 4. The elbow-point based method offers one systematic way to determine the cutoff used for calling CNAs.

(**a**) The elbow method for determining the cutoff of |log2 copy ratio| was used in calling AS for individual cancer types (exemplified by esophagogastric cancer here). The AS for each patient with different calling cutoffs are shown in black curves. The mean value of all patients is shown in the red curve. The mean elbow point is shown with 95% confidence intervals, which are calculated using 1,000-replicate bootstrapping. (**b**) The elbow point values of the cutoff of |log2 copy ratio| in calculating AS in individual cancer types. (**c**) Comparison of HRs using $AS_{0.1}$ or AS_{EP} in a multivariate Cox model with TMB (binned at the 80th percentile) and ICB drug class. Difference of mean HRs of $AS_{0.1}$ and AS_{EP} and paired Wilcoxon test p value are displayed. Wald p values for HRs of AS_{EP} in individual cancer types are displayed at the right side of the plot. (**d**)

Univariable Kaplan-Meier survival analysis and multivariable survival analysis using Cox proportional hazards regression of overall survival with AS calculated using cancer-type-specific elbow point based CNA calling cutoff (AS_{EP}; binned at the 30th percentile), TMB (binned at the 80th percentile), and ICB drug class. Wald p values are displayed. In panel b, the bars represent 95% confidence intervals of the elbow point values calculated using a 1000-replicate bootstrapping. In panel c, the upper and lower boundaries signify the first and third quartiles, correspondingly, while the central line denotes the median. Whiskers stretch to the most distant data points not classified as outliers (within 1.5 times the interquartile range), and outliers are illustrated as points above and below the box-and-whisker diagram. In panel d, squares positioned at midpoints symbolize point estimates of HRs, and the accompanying bars indicate 95% confidence intervals. The data are from the Samstein et al. cohort [1].

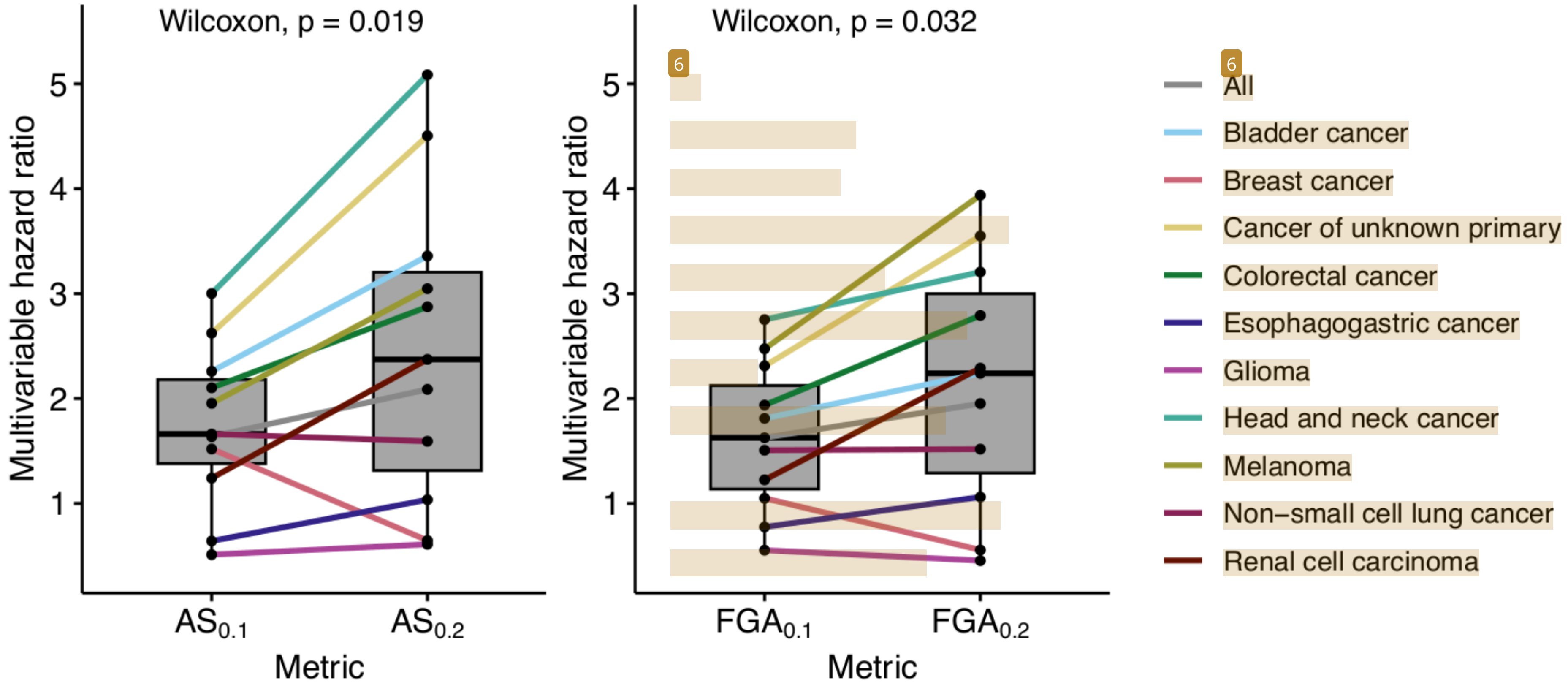
Wilcoxon, p = 0.019
Multivariable hazard ratio
5
4
3
2
1
AS0.1
AS0.2
Metric
Wilcoxon, p = 0.032
Multivariable hazard ratio
5
4
3
2
1
FGA0.1
FGA0.2
Metric
All
Bladder cancer
Breast cancer
Cancer of unknown primary
Colorectal cancer
Esophagogastric cancer
Glioma
Head and neck cancer
Melanoma
Non−small cell lung cancer
Renal cell carcinoma

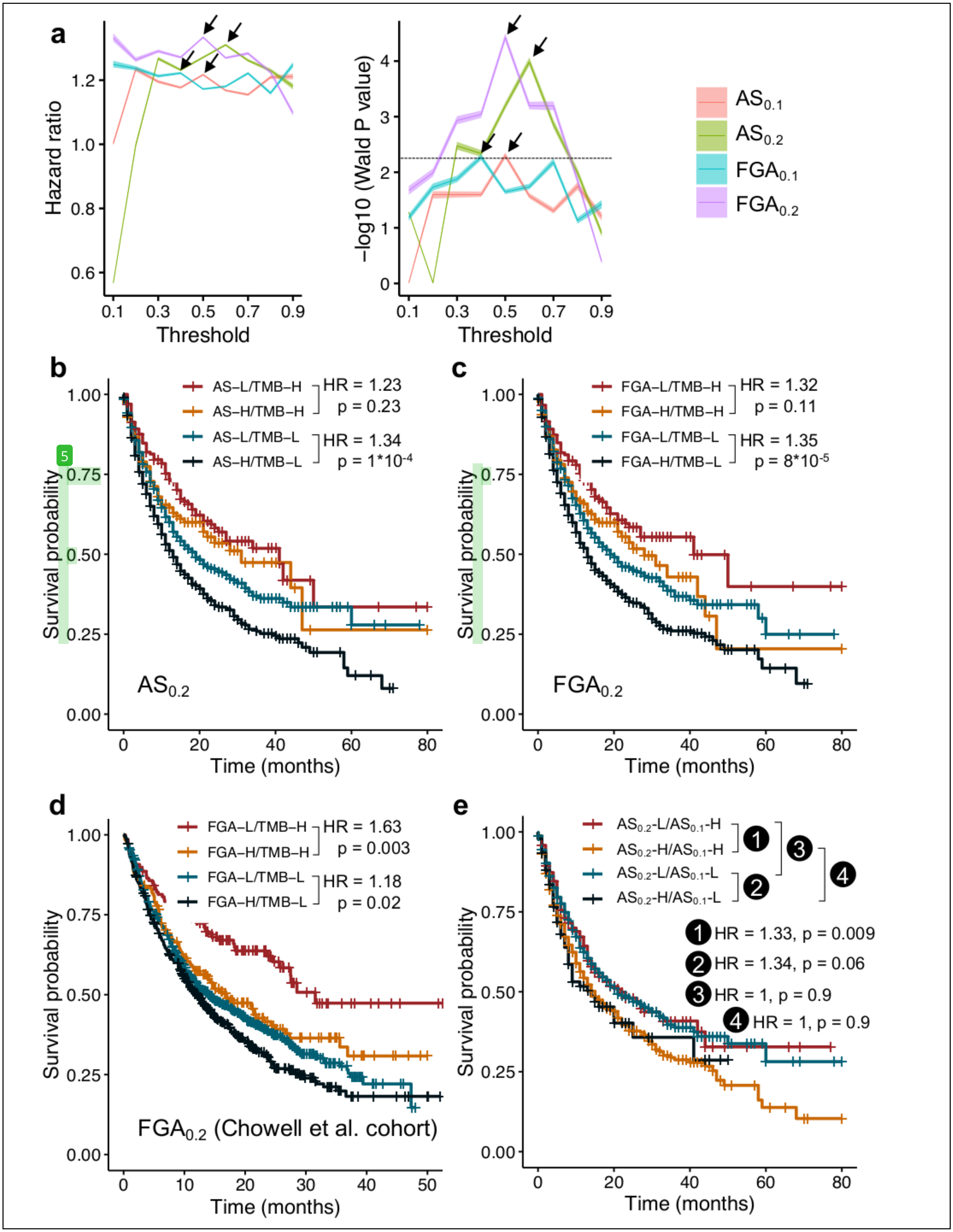
a
Hazard ratio
Threshold
−log10 (Wald P value)
AS0.1
AS0.2
FGA0.1
FGA0.2
b
AS−L/TMB−H
AS−H/TMB−H
AS−L/TMB−L
AS−H/TMB−L
HR = 1.23
p = 0.23
HR = 1.34
p = 1*10-4
Survival probability
Time (months)
AS0.2
c
FGA−L/TMB−H
FGA−H/TMB−H
FGA−L/TMB−L
FGA−H/TMB−L
HR = 1.32
p = 0.11
HR = 1.35
p = 8*10-5
FGA0.2
d
HR = 1.63
p = 0.003
HR = 1.18
p = 0.02
FGA0.2 (Chowell et al. cohort)
e
AS0.2-L/AS0.1-H
AS0.2-H/AS0.1-H
AS0.2-L/AS0.1-L
AS0.2-H/AS0.1-L
1 HR = 1.33, p = 0.009
2 HR = 1.34, p = 0.06
3 HR = 1, p = 0.9
4 HR = 1, p = 0.9

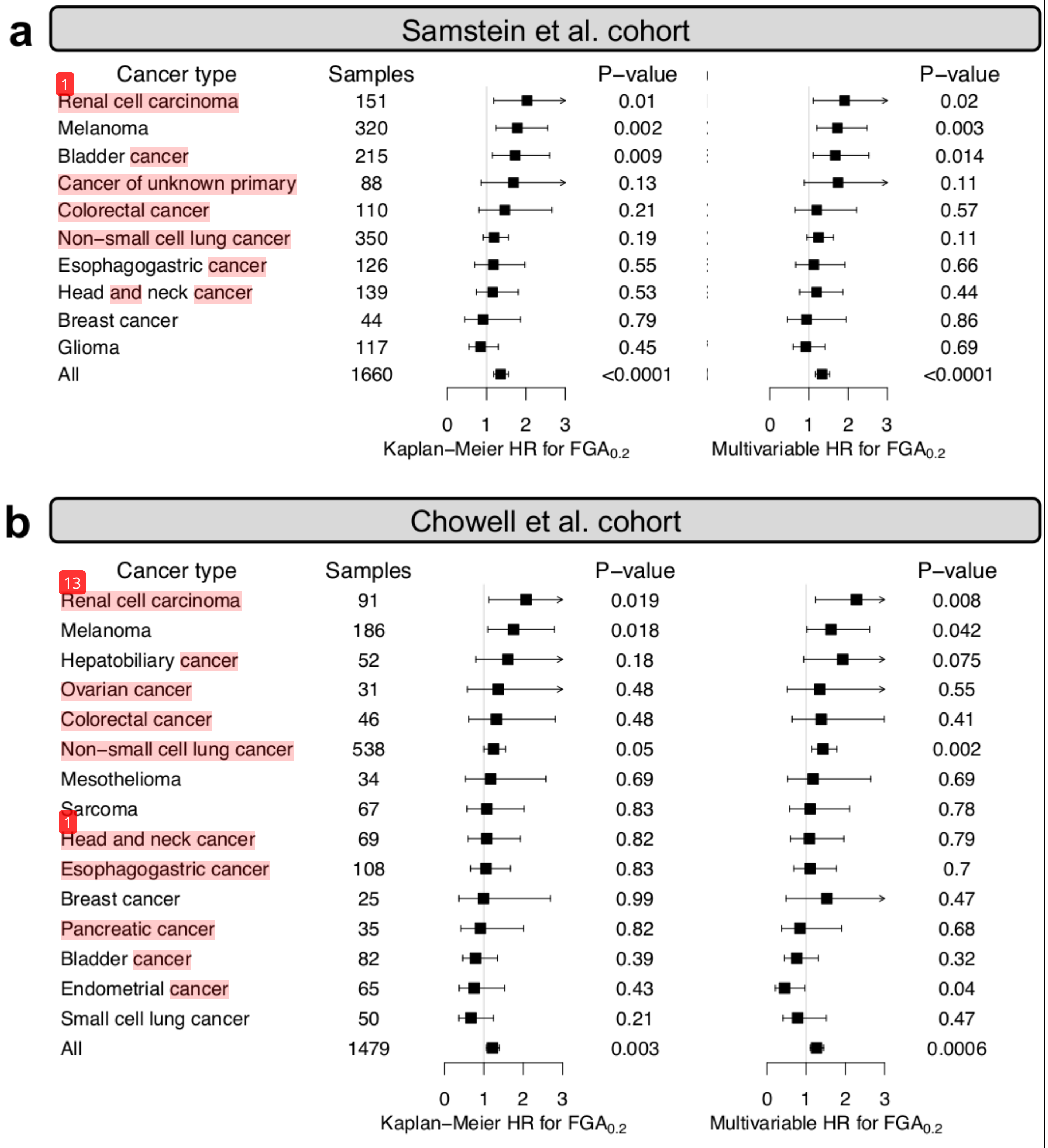
a
Samstein et al. cohort
Cancer type
Samples
P−value
P−value
Renal cell carcinoma 151 0.01 0.02
Melanoma 320 0.002 0.003
Bladder cancer 215 0.009 0.014
Cancer of unknown primary 88 0.13 0.11
Colorectal cancer 110 0.21 0.57
Non−small cell lung cancer 350 0.19 0.11
Esophagogastric cancer 126 0.55 0.66
Head and neck cancer 139 0.53 0.44
Breast cancer 44 0.79 0.86
Glioma 117 0.45 0.69
All 1660 <0.0001 <0.0001
0 1 2 3
0 1 2 3
Kaplan−Meier HR for FGA0.2
Multivariable HR for FGA0.2
b
Chowell et al. cohort
Cancer type
Samples
P−value
P−value
Renal cell carcinoma 91 0.019 0.008
Melanoma 186 0.018 0.042
Hepatobiliary cancer 52 0.18 0.075
Ovarian cancer 31 0.48 0.55
Colorectal cancer 46 0.48 0.41
Non−small cell lung cancer 538 0.05 0.002
Mesothelioma 34 0.69 0.69
Sarcoma 67 0.83 0.78
Head and neck cancer 69 0.82 0.79
Esophagogastric cancer 108 0.83 0.7
Breast cancer 25 0.99 0.47
Pancreatic cancer 35 0.82 0.68
Bladder cancer 82 0.39 0.32
Endometrial cancer 65 0.43 0.04
Small cell lung cancer 50 0.21 0.47
All 1479 0.003 0.0006
0 1 2 3
0 1 2 3
Kaplan−Meier HR for FGA0.2
Multivariable HR for FGA0.2

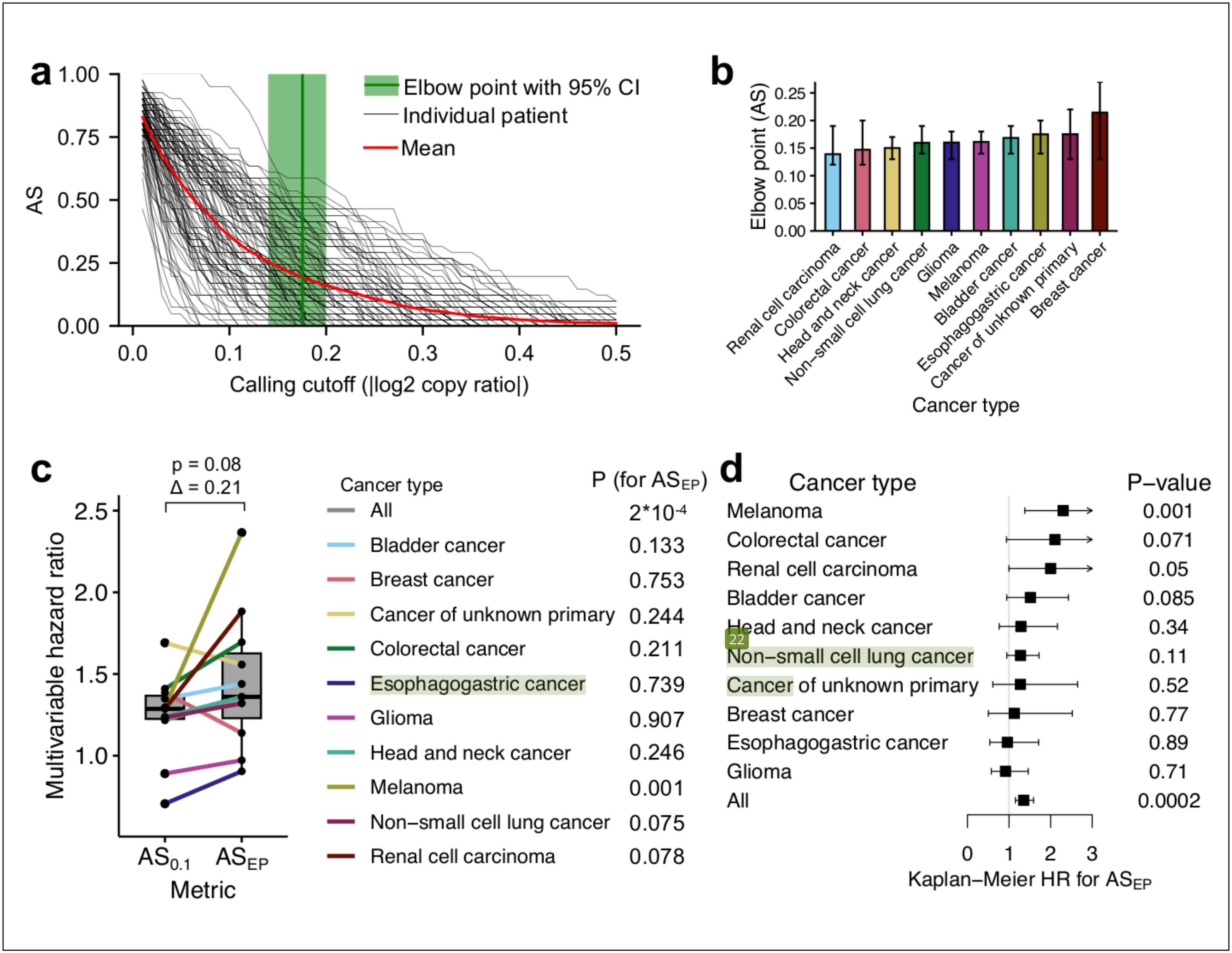
a
AS
1.00
0.75
0.50
0.25
0.00
0.0
0.1
0.2
0.3
0.4
0.5
Calling cutoff (|log2 copy ratio|)
Elbow point with 95% CI
Individual patient
Mean
b
Elbow point (AS)
0.25
0.20
0.15
0.10
0.05
0.00
Renal cell carcinoma
Colorectal cancer
Head and neck cancer
Non−small cell lung cancer
Glioma
Melanoma
Bladder cancer
Esophagogastric cancer
Cancer of unknown primary
Breast cancer
Cancer type
c
p = 0.08
Δ = 0.21
Multivariable hazard ratio
2.5
2.0
1.5
1.0
AS0.1
ASEP
Metric
Cancer type
P (for ASEP)
All 2*10-4
Bladder cancer 0.133
Breast cancer 0.753
Cancer of unknown primary 0.244
Colorectal cancer 0.211
Esophagogastric cancer 0.739
Glioma 0.907
Head and neck cancer 0.246
Melanoma 0.001
Non−small cell lung cancer 0.075
Renal cell carcinoma 0.078
d
Cancer type
P−value
Melanoma 0.001
Colorectal cancer 0.071
Renal cell carcinoma 0.05
Bladder cancer 0.085
Head and neck cancer 0.34
22
Non−small cell lung cancer 0.11
Cancer of unknown primary 0.52
Breast cancer 0.77
Esophagogastric cancer 0.89
Glioma 0.71
All 0.0002
0
1
2
3
Kaplan−Meier HR for ASEP

tiangen.pdf

ORIGINALITY REPORT

49%
SIMILARITY INDEX

PRIMARY SOURCES

1	assets.researchsquare.com<br>Internet	1566 words — 21%
2	www.researchsquare.com<br>Internet	1360 words — 18%
3	www.nature.com<br>Internet	290 words — 4%
4	Tiangen Chang, Yingying Cao, Eldad Shulman, Alejandro Schäffer, Uri Ben-David, Eytan Ruppin. "Optimizing cancer immunotherapy response prediction by tumor aneuploidy score and fraction of copy number alterations", Research Square Platform LLC, 2023<br>Crossref Posted Content	106 words — 1%
5	www.biorxiv.org<br>Internet	93 words — 1%
6	Liam F. Spurr, Ralph R. Weichselbaum, Sean P. Pitroda. "Tumor aneuploidy predicts survival following immunotherapy across multiple cancers", Nature Genetics, 2022<br>Crossref	59 words — 1%
7	Guangxian Meng, Xiaowei Liu, Tian Ma, Desheng Lv, Ge Sun. "Predictive value of tumor mutational	42 words — 1%

burden for immunotherapy in non-small cell lung cancer: A systematic review and meta-analysis", PLOS ONE, 2022
Crossref

---

8 summit.sfu.ca — 39 words — 1%
Internet

---

9 Deepesh Kumar Gupta, Jian Du, Siamak A. Kamranvar, Staffan Johansson. "Tension-induced cytokinetic abscission in human fibroblasts", Oncotarget, 2018 — 24 words — < 1%
Crossref

---

10 github.com — 21 words — < 1%
Internet

---

11 www.science.gov — 16 words — < 1%
Internet

---

12 Tiangen Chang, Yingying Cao, Eldad D. Shulman, Alejandro A. Schäffer, Eytan Ruppin. "Fraction of copy-number alterations significantly predicts survival following immunotherapy in a few cancers", Cold Spring Harbor Laboratory, 2022 — 13 words — < 1%
Crossref Posted Content

---

13 www.frontiersin.org — 13 words — < 1%
Internet

---

14 jitc.bmj.com — 10 words — < 1%
Internet

---

15 Bastien Nguyen, Christopher Fong, Anisha Luthra, Shaleigh A. Smith et al. "Genomic characterization of metastatic patterns from prospective clinical sequencing of 25,000 patients", Cell, 2022 — 8 words — < 1%
Crossref

16 Demi L. Pink, Orathai Loruthai, Robert M. Ziolek, Ann E. Terry, David J. Barlow, M. Jayne Lawrence, Christian D. Lorenz. "Interplay of lipid and surfactant: Impact on nanoparticle structure", Journal of Colloid and Interface Science, 2021 — 8 words — < 1%

Crossref

17 Hendrik J. Kuiken, Sabin Dhakal, Laura M. Selfors, Chandler M. Friend et al. "Clonal populations of a human TNBC model display significant functional heterogeneity and divergent growth dynamics in distinct contexts", Oncogene, 2021 — 8 words — < 1%

Crossref

18 John J. Wallbillich, Robert T. Morris, Rouba Ali-Fehmi. "Comparing mutation frequencies for homologous recombination genes in uterine serous and high-grade serous ovarian carcinomas: A case for homologous recombination deficiency testing in uterine serous carcinoma", Gynecologic Oncology, 2020 — 8 words — < 1%

Crossref

19 Qingyu Xu, Eva Altrock, Nanni Schmitt, Alexander Streuer et al. "In Silico Pan-Cancer Analysis Reveals Prognostic Role of the Erythroferrone (ERFE) Gene in Human Malignancies", International Journal of Molecular Sciences, 2023 — 8 words — < 1%

Crossref

20 Susan C. Scott, Xiaoshan M. Shao, Noushin Niknafs, Archana Balan et al. "Sex-specific differences in immunogenomic features of response to immune checkpoint blockade", Frontiers in Oncology, 2022 — 8 words — < 1%

Crossref

21 www.cell.com — 8 words — < 1%

Internet

22	www.csnpharm.com<br>Internet	8 words — < 1%
23	www.diva-portal.org<br>Internet	8 words — < 1%
24	Anisha Luthra, Brooke Mastrogiacomo, Shaleigh A. Smith, Debyani Chakravarty, Nikolaus Schultz, Francisco Sanchez‐Vega. "Computational methods and translational applications for targeted next‐generation sequencing platforms", Genes, Chromosomes and Cancer, 2022<br>Crossref	6 words — < 1%
25	Liangliang Lei, Nannan Li, Pengfei Yuan, Dechun Liu. "A new risk model based on a 11-m6A-related lncRNA signature for predicting prognosis and monitoring immunotherapy for gastric cancer", BMC Cancer, 2022<br>Crossref	6 words — < 1%

EXCLUDE QUOTES	OFF	EXCLUDE SOURCES	OFF
EXCLUDE BIBLIOGRAPHY	OFF	EXCLUDE MATCHES	OFF
